# Novel System for Measuring Tension Force in Eyeball Movement

**DOI:** 10.3390/bios15120769

**Published:** 2025-11-25

**Authors:** Jae Yun Sung, Ju Mi Kim, Il Doh, Yeon-Hee Lee

**Affiliations:** 1 Department of Ophthalmology, Chungnam National University Sejong Hospital, Sejong 30099, Republic of Korea; sunsung84@cnuh.co.kr; 2 Department of Ophthalmology, Chungnam National University College of Medicine, Daejeon 35015, Republic of Korea; 3 Department of Ophthalmology, Daejeon St. Mary’s Hospital, College of Medicine, The Catholic University of Korea, Seoul 34943, Republic of Korea; jjukkum2@cmcnu.or.kr; 4 Department of Applied Measurement Engineering, University of Science and Technology (UST), Daejeon 34113, Republic of Korea; 5 Medical Metrology Group, Division for Biomedical Metrology, Korea Research Institute of Standards and Science (KRISS), Daejeon 34113, Republic of Korea; 6 Department of Ophthalmology, Chungnam National University Hospital, Daejeon 35015, Republic of Korea

**Keywords:** passive tension of ocular rotation, forced duction test, ocular motility disorder

## Abstract

Accurate assessment of extraocular muscle mechanics is crucial for diagnosing and treating ocular motility disorders, yet current methods, such as the forced duction test, rely on subjective tactile sensation and gross visual observation. To overcome the limitations of subjectivity and the impracticality of previous quantitative devices, we developed a novel biosensing system capable of simultaneously and objectively measuring passive ocular tension and rotation angle during forced duction. The system integrates custom-engineered surgical forceps equipped with dual strain gauges and an infrared video camera that precisely tracks pupil displacement to calculate real-time rotation angle. We clinically validated this system in a prospective study involving 10 patients (20 eyes) with intermittent exotropia, with measurements performed under general anesthesia. Reliable tension–angle curves were successfully obtained in all cases without complications. Passive tension increased progressively with ocular rotation, following a linear-parabolic trajectory up to 40°. The mean duction force of the medial and lateral rectus muscles showed comparable symmetry. This lightweight, practical, and objective biosensing system offers a reliable tool for quantifying ocular mechanics, with the potential to enhance diagnostic accuracy, enable individualized surgical planning, and support fundamental research in ocular motility disorders.

## 1. Introduction

Ocular motility is an essential function for visual perception, enabling the coordinated movement of both eyes to achieve binocular single vision [1,2]. The impairment of mobility in even one eye can disrupt this coordination, leading to diplopia, a highly debilitating symptom often causing more distress than the loss of vision in one eye [3]. The assessment of mechanical properties, such as passive resistance, active force, and range of motion, provides fundamental information for the diagnosis and treatment of ocular motility disorders and serves as a crucial dataset for clinical research.

Despite its importance, the clinical techniques currently used to evaluate these properties remain rudimentary. The assessment of passive tension and active force typically relies on the subjective, tactile sensation of the examiner during procedures like the FDT [4,5]. Similarly, the range of motion is often estimated by gross visual observation. To overcome these limitations and enable objective quantification, several measurement techniques have been proposed. Early approaches included the use of perilimbal suction caps [4], spring gauge dynamometers [6], and strain gauge–equipped forceps combined with ultrasonography [7]. Subsequent developments employed strain gauge transducers attached to the sclera with a loop [8], and more recent systems have integrated locking forceps with load cells and biaxial tilt sensors [9,10,11] to facilitate compact, direct measurements of extraocular muscle tension. Additional devices for evaluating active tension and intraoperative tensile strength have also been introduced [12,13]. In this study, we introduce a novel device engineered to quantitatively and simultaneously measure passive tension and the corresponding rotation angle during forced duction. We aim to report the design, validation, and clinical application of this system in strabismus patients, thereby providing a reliable method for characterizing the mechanical resistance profile of the eyeball rotation.

## 2. Materials and Methods

### 2.1. System Overview

**Figure 1:**
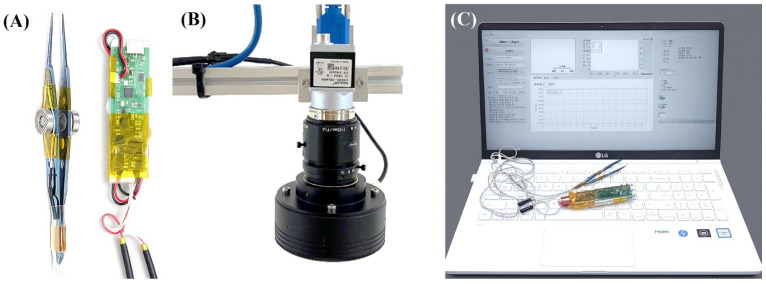
Overview of the biosensing system for quantitative measurement of passive ocular tension and rotation angle. (**A**) Passive tension measurement subsystem: forceps with integrated strain gauges connected to a custom-designed printed circuit board for signal acquisition and processing. (**B**) Rotation angle measurement subsystem: an infrared camera captured high-contrast images of the eye, and a custom LabVIEW application performed image processing and angle calculations. (**C**) Display unit: processed signals were transmitted via Bluetooth for real-time monitoring and data logging.

**Figure 2:**
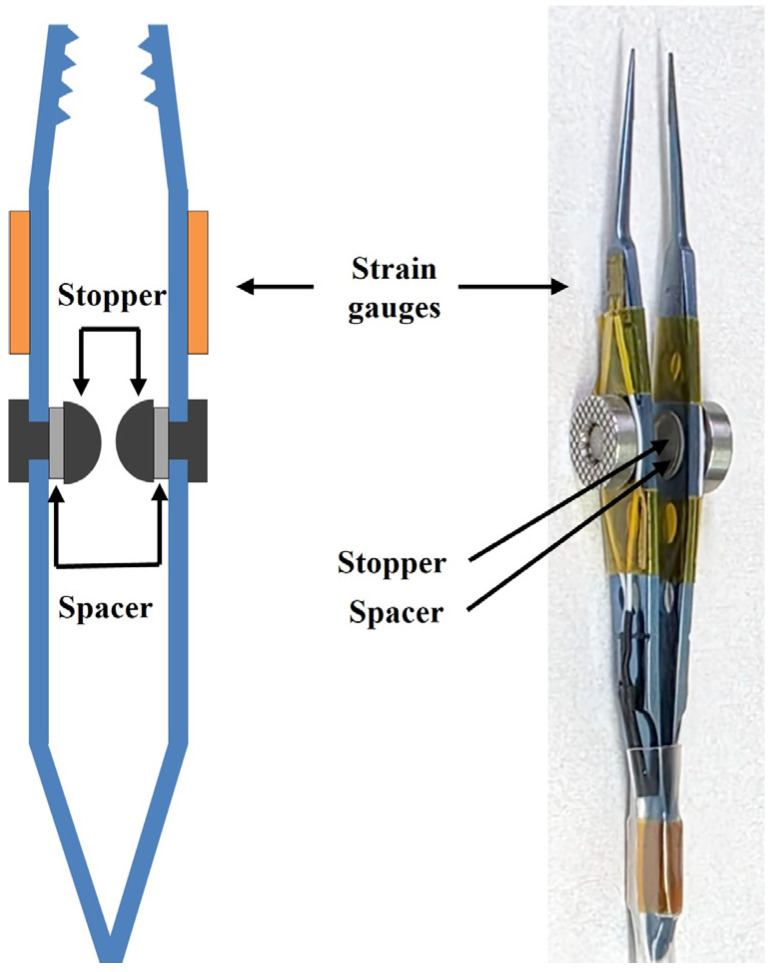
Design of the passive tension measurement unit. Two miniature strain gauges were affixed to the outer surfaces of the forceps to measure applied force, and a mechanical stopper was incorporated between the arms to minimize noise from variability of gripping force, ensuring a constant clamping pressure on the conjunctiva regardless of the examiner’s hand pressure.

The measurement system (Figure 1) comprises two primary synchronized subsystems: (1) a passive tension measurement unit consisting of custom-engineered forceps (Figure 1A) and (2) an ocular rotation angle measurement unit based on an infrared video camera (Figure 1B). During a procedure, the examiner grasps the conjunctiva with the forceps and manually rotates the fully relaxed eye of the subject. The forceps continuously measure the passive tension, while the camera system simultaneously tracks the pupil’s movement to calculate the real-time rotation angle. Both data streams are transmitted to a host PC, where custom software plots a tension-versus-angle graph, providing an objective profile of the eye’s mechanical properties (Figure 1C).

### 2.2. Passive Tension Measurement Subsystem

#### 2.2.1. Forceps with Integrated Strain Gauges

To quantify the force applied during the FDT, two miniature strain gauges (dimensions: 3.0 mm × 4.5 mm; sensing grid: 1.0 mm × 1.6 mm) were bonded to the outer surfaces of the opposing arms of a standard pair of surgical forceps. Commercial strain gauges (FLA-1-350, TMI, Tokyo, Japan) were used in the design. The assembled system, including the bonding interface, demonstrated stable performance without noticeable hysteresis, confirming the reliability of the attachment for force measurement. High-resistance (350 Ω) gauges were selected to enhance signal stability. The two gauges were configured in a Half-Wheatstone Bridge circuit. This configuration allows for differential measurement: when the forceps grip the conjunctiva, both gauges experience tensile strain. However, when the eye is subsequently moved, the gauge on the leading arm undergoes reduced strain (resistance decrease), while the gauge on the trailing arm experiences increased tensile strain (resistance increase). The differential signal between the two is directly proportional to the net force applied to the eye. To eliminate measurement bias arising from variations in gripping force, a mechanical stopper was installed between the forceps arms, ensuring a constant clamping pressure on the conjunctiva regardless of the examiner’s hand pressure Additionally, a 0.1 mm thick spacer was inserted between the stopper and the forceps arms, to allow the initial gripping gap to be adjusted according to the subject’s conjunctival thickness (Figure 2). Once the stopper components make contact, they form a mechanical hard stop that maintains a consistent clamping force on the conjunctiva, regardless of the examiner’s applied hand pressure. This design standardizes the gripping force while accommodating small variations in conjunctival thickness.

#### 2.2.2. Signal Processing and Wireless Transmission

The electrical signals from the strain gauges were processed by a custom-designed Printed Circuit Board (PCB). The PCB, powered by a self-contained lithium-ion battery, includes a differential amplifier and an analog-to-digital converter to process the output from the Wheatstone bridge. The custom PCB incorporated a 24-bit analog-to-digital converter with a sampling rate of 80 Hz. To prevent the PCB’s weight from interfering with the sensitive force measurements, it was separated and connected to the forceps via a flexible, 1-m-long cable. The processed signal is digitized and transmitted wirelessly to the host PC via Bluetooth communication, enabling real-time monitoring and data logging.

#### 2.2.3. Calibration

**Figure 3:**
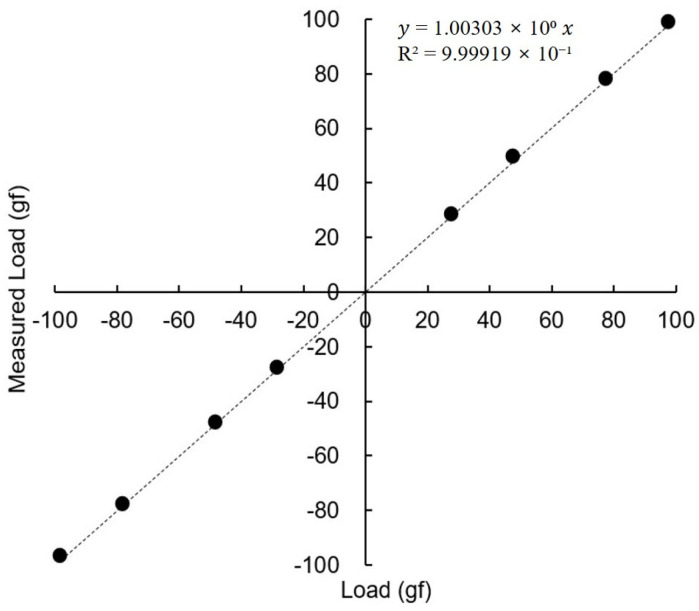
Calibration of the passive tension measurement unit using standard weights. The device demonstrated excellent linearity and accuracy across the tested range.

The tension measurement forceps were calibrated in a laboratory setting. With the forceps held horizontally, a series of standard weights (up to 100 gf) were suspended from the tip. The device demonstrated excellent linearity and accuracy across the tested range, confirming its suitability for clinical measurements (Figure 3).

### 2.3. Ocular Rotation Angle Measurement Subsystem

#### 2.3.1. Hardware and Image Acquisition

An infrared (IR) camera (acA2000-165um USB 3.0, Basler AG, Ahrensburg, Germany) was used to capture high-contrast images of the subject’s eye. To ensure consistent and clear pupil definition and to prevent shadows, the eye was illuminated by a ring-shaped IR LED light source mounted on the camera lens, supplemented by two additional IR sources.

#### 2.3.2. Image Processing and Angle Calculation

All image processing and angle calculations were performed using a custom software application developed in LabVIEW (National Instruments, USA). The algorithm executed the following steps in real-time for each video frame. First, the captured image is converted to an 8-bit grayscale format. Next, a binary threshold is applied to segment the bright pupil from the surrounding iris. A particle analysis algorithm then identifies the pupil as the largest contiguous blob and calculates its geometric centroid, which yields the pupil center’s coordinates (x, y) in pixels. Finally, the pixel displacement of the pupil’s center is converted into a rotational angle using a geometric model. This conversion requires the subject’s specific ocular radius (r). To preserve a constant pixel scale, the distance between the camera and the eye was fixed at 50 cm for all measurements, maintaining a consistent pixel-to-angle conversion and ensuring calibration validity.

### 2.4. Subject-Specific Pre-Measurement Procedures

#### 2.4.1. Measurement of Individual Ocular Radius (R)

**Figure 4:**
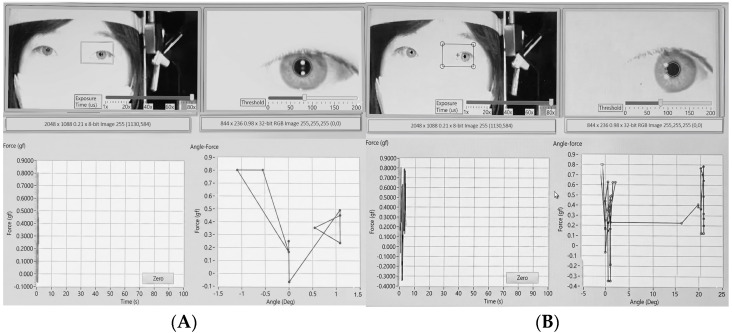
Determination of individual ocular radius. The subject was instructed to fixate first on the central target (**A**) and then on a peripheral target (**B**) located at a known angle (20°) on the wall of examining room. The software recorded the center of pupil’s pixel displacement between the two fixation points. By substituting the known angle (20°) and the measured displacement into the conversion formula, the subject-specific ocular radius (r) was calculated.

Prior to the passive tension measurement, each subject’s ocular radius was determined. The subject was instructed to fixate first on the central target (Figure 4A) and then on a peripheral target located at a known angle (20°) on the wall of examining room (Figure 4B). The software recorded the pupil’s pixel displacement between the two fixation points. By substituting the known angle (20°) and the measured displacement into the conversion formula, the subject-specific ocular radius (r) was calculated and stored (Figure 4).

#### 2.4.2. Determination of the Zero-Degree Reference Point

To establish a reliable zero-degree reference, the subject’s kappa distance was first defined while they were awake and fixating on a distant target. For this procedure, the subject was instructed to focus on a central target, and an IR image was captured using a camera positioned between them and the target. The distance between the center of the pupil and the corneal reflex in this captured image was defined as the kappa distance.

Under general anesthesia, the eye naturally deviates to a passive resting position. Therefore, before beginning the measurements, the examiner manually rotated the eye while observing a live video feed. This adjustment continued until the distance between the pupil center, and the corneal reflex precisely matched the previously captured kappa distance. This aligned position was then established as the 0° reference point in the software.

### 2.5. Clinical Measurement Protocol

This prospective study included patients who were scheduled to receive surgery for intermittent exotropia at Chungnam National University Hospital. The study protocol was approved by the Institutional Review Board of Chungnam National University Hospital (IRB number: CNUH 2021-07-059) and adhered to the tenets of the Declaration of Helsinki. Written informed consent was obtained from all participants.

All measurements were performed under general anesthesia to ensure complete relaxation of the extraocular muscles. Standard ASA (American Society of Anesthesiologists) monitoring was maintained throughout the procedure. General anesthesia was induced with intravenous propofol (1–2 mg/kg) and rocuronium (0.6 mg/kg) to facilitate endotracheal intubation. After intubation, anesthesia was maintained with sevoflurane inhalation in combination with a continuous intravenous infusion of remifentanil (0.05–0.2 μg/kg/min). To minimize the influence of residual muscle tone, all measurements were performed under deep neuromuscular blockade, confirmed by a stable depth of anesthesia (BIS 40–60). Measurements were obtained only after a hemodynamic stabilization period, and any data acquired outside these criteria were excluded.

**Figure 5:**
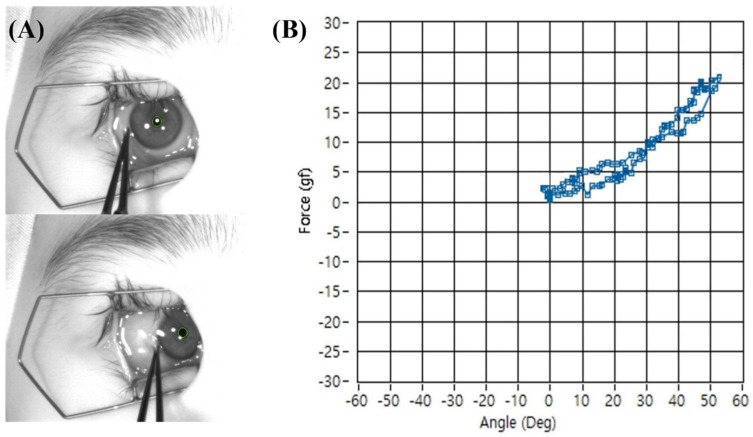
Representative photograph of passive tension measurement during adduction of the right eye. (**A**) The conjunctiva near the limbus was grasped with forceps, and the eye was rotated horizontally past 40°. (**B**) The software simultaneously recorded passive tension and rotation angle in real time, producing a mechanical resistance profile. The difference in tension measurements at each angle during outward and return movements generated a separate trajectory (hysteresis loop), reflecting the viscoelastic properties of the eyeball rotation.

The subject was placed in a supine position, and their head was stabilized to face directly upwards. A laser level was used to position the camera rig perpendicular to the corneal apex. The subject-specific ocular radius (r) was entered into the software. After placing a lid speculum, the examiner grasped the conjunctiva near the limbus with the forceps. During measurement, the examiner was instructed to move the forceps tangentially along the curvature of the ocular surface, approximately perpendicular to the ocular radius at the point of contact, to minimize artifacts from off-axis forces. While observing the live video feed, the eye was rotated to match the pre-determined angle kappa reference, and this position was set as 0°. The examiner then smoothly rotated the eyeball horizontally (adduction and abduction) past 40° in each direction (Figure 5A) and then rotated the eyeball back to zero position. To minimize the effect of tissue hysteresis, the examiner rotated the eyeball slowly keeping constant rotation speed (10°/s). The software simultaneously recorded the passive tension from the forceps and the calculated rotation angle, generating a real-time plot of the mechanical resistance profile for each eye (Figure 5B). Each rotation angle had two measurements taken during the rotating out and rotating back processes. The mid-point of the two measurements was set as the true passive tension force and used in the study to minimize the effect of tissue hysteresis.

## 3. Results

A total of 10 patients (20 eyes) were included in this study. The mean age was 9.0 ± 1.9 years (range, 6–13 years), and 40% of patients were male. Passive tension–angle curves were successfully obtained in all cases. The average passive tension increased gradually with ocular rotation, displaying linear-parabolic trajectory up to 40° in both adduction and abduction, with a steeper rise near the endpoint. Representative plots are shown in Figure 5. The mean passive duction force of the lateral rectus muscle during adduction was 3.3 ± 1.2, 5.5 ± 1.8, 8.6 ± 2.7, and 13.4 ± 3.8 g at 10°, 20°, 30°, and 40°, respectively. The corresponding mean passive duction force of the medial rectus muscle during abduction was 3.1 ± 2.1, 5.6 ± 2.4, 8.7 ± 2.7, and 14.3 ± 3.3 g, respectively. All procedures were completed without complications such as conjunctival or corneal injury.

## 4. Discussion

In this study, we successfully developed a novel biosensing system for the quantitative and simultaneous measurement of passive ocular tension and rotation angle. This system was designed to overcome the distinct limitations of previous approaches: the subjectivity of the conventional FDT and the impracticality of earlier quantitative devices. By providing objective data, this system has the potential to introduce a new paradigm for the diagnosis and treatment of ocular motility disorders.

Various quantitative devices have been introduced to measure extraocular muscle tension. Scott et al. [14] developed torque wrench-modified forceps to quantify both active and passive forces, while Stephens and Reinecke [4] used a perilimbal suction cup connected to a force–displacement transducer to assess passive tension. Rosenbaum and Mey [6] applied a spring dynamometer to the limbal conjunctiva via self-retaining Pierce forceps, and Metz and Cohen [8,15] developed a strain gauge transducer usable under general anesthesia. Although these methods demonstrated the feasibility of quantitative measurement, they were cumbersome, invasive, and time-consuming, limiting their routine clinical applicability. More recently, Shin et al. [9,10,11] introduced a compact extraocular muscle tension-measuring device that combined locking forceps, a load cell, and a biaxial tilt sensor. While this system represented progress toward clinical applicability, it was still relatively bulky and heavy compared with our newly developed device. Our forceps weigh only 17 g, providing improved ergonomics and ease of use. Although prior reports did not specify weight or dimensions, their larger and more complex designs suggest reduced practicality. Miniaturization and weight reduction were key design goals to enhance feasibility and user comfort.

Our system was designed to address these limitations. The ocular tissues, particularly the cornea and conjunctiva, are delicate and sensitive to physical irritation and trauma. When handling the eye of a patient lying in supine position, the weight of the surgical instrument is a critical safety factor. A heavy instrument increases the risk of being dropped, which could cause severe corneal injury from the sharp forceps tips. Moreover, a cumbersome instrument impairs the fine control necessary for the procedure and makes it difficult for the examiner to perceive inadvertent tears of conjunctiva and subtle changes in tissue resistance. We therefore based our design on a lightweight, standard surgical forceps, adding only the minimal mass of the strain gauges to ensure the instrument remained ergonomic and safe. This design differs from previous approaches which employed locking forceps. Although a locking mechanism may reduce operator fatigue when used with a lightweight instrument, it can pose a substantial hazard with heavier devices or improper handling, significantly increasing the risk of conjunctival tearing. We deliberately selected a non-locking design to mitigate this risk. A limitation of non-locking forceps, however, is that variable gripping force can introduce signal noise. To address this, we incorporated an adjustable stopper between the arms, which mechanically maintains constant conjunctival pressure independent of examiner hand force, thereby improving data reliability and preventing excessive pressure or tissue injury.

Measurements obtained with our novel system showed that passive tension increased gradually with ocular rotation, following a near-linear profile with a steeper rise near the endpoint. This behavior is consistent with the biomechanical properties of extraocular muscles, in which resistance increases as the elastic limit is approached. The measured tension values reflect passive elastic resistance under deep anesthesia, representing the passive stiffness of the muscle–tendon–connective tissue complex rather than active contractile force [16]. These passive forces are thought to represent the baseline viscoelastic load opposing ocular rotation but do not directly correspond to the functional contractile strength of the extraocular muscles in vivo, which depends on neural activation and dynamic force generation. Passive duction forces of the lateral and medial rectus muscles were comparable across adduction and abduction, suggesting that in patients with intermittent exotropia, passive tension of ocular rotation remains within a normal physiological range despite ocular misalignment. These findings are consistent with prior reports using experimental force-measuring devices, which also demonstrated symmetric profiles in comitant strabismus. Earlier studies have shown that the medial rectus muscle can generate greater force than the lateral rectus muscle under active or mixed active–passive conditions due to differences in neural activation and motor unit recruitment [7,17]. In contrast, our measurements were obtained under deep general anesthesia, reflecting purely passive viscoelastic resistance with suppressed active contractile force. Under these passive conditions, stiffness values are known to be similar between the medial rectus muscle and lateral rectus muscle, consistent with prior intraoperative findings [6,17,18]. Notably, Rosenbaum and Myer [6], using a spring gauge dynamometer under general anesthesia, reported mean forces of 6, 10, and 16 g at 15°, 30°, and 45°, corresponding to 0.3–0.4 g per degree of rotation. In our study, the mean tension was 0.27–0.36 g per degree, closely matching their values.

The clinical utility of this system is expected to be extensive. First, it enables objective FDT for the diagnosis of restrictive or paretic strabismus. Furthermore, the system is not limited to measuring passive tension; it can be extended to measure the active force generated by a conscious patient attempting to move their eye, providing a quantitative force generation test. This would yield an invaluable metric for assessing the degree of muscle paresis. Second, this system could improve the predictability of strabismus surgery. It is a common experience among surgeons that identical surgical procedures can yield highly variable outcomes in different patients. This is likely because conventional surgical planning does not account for the unique biomechanical properties of each patient’s eye. By measuring a patient’s passive tension profile preoperatively, our system can characterize these individual biomechanical signatures, such as muscle stiffness or tissue compliance. This data could facilitate customized surgery, where surgical amounts are tailored to the patient’s specific mechanics, potentially leading to more consistent and successful outcomes. Finally, the system may serve as a valuable tool for both fundamental and clinical research in oculomotor physiology and biomechanics, enabling comparative studies of mechanical properties across patient populations (e.g., thyroid eye disease vs. congenital fibrosis) and quantitative evaluation of novel therapeutic interventions.

This study has several limitations. The measurements were obtained in a relatively small cohort limited to patients with intermittent exotropia, which may limit the generalizability of the findings. In addition, the current system is restricted to horizontal measurements, and future work should extend its capability to vertical and torsional directions. Vertical measurements are particularly constrained because, at extreme rotations, the eyelid obscures the pupil center and prevents reliable tracking. Technical refinements will therefore be required; however, the biomechanical principles demonstrated in the horizontal rectus muscles are likely applicable to the vertical rectus muscles as well. Finally, intra- and inter-observer reproducibility could not be assessed, as repeated intraoperative measurements were restricted to minimize effect of the experiment and anesthesia duration. Despite these limitations, we believe that our instrument provides a practical means of obtaining quantitative measurements of forced duction. In future studies, we plan to expand the cohort to include patients with restrictive and paretic strabismus to compare their tension–angle characteristics with those of normal muscles. This may enable the establishment of reference ranges and potential thresholds that could assist in differentiating normal from pathologic extraocular muscle conditions in clinical practice. Large-scale clinical studies are also needed to compare passive muscle tension across normal individuals and to analyze the correlations between these biomechanical measurements and surgical outcomes. Although the current system operates under intraoperative conditions, it serves as a proof of concept establishing a quantitative framework for measuring the passive tension of ocular rotation. Future refinements may enable miniaturized and wearable implementations for dynamic and noninvasive monitoring. Recent advances in smart contact lens-based biosensing have demonstrated the potential for continuous ocular health monitoring through innovations in material design, sensor integration, and wireless power delivery [19,20]. These developments highlight the long-term direction toward continuous, minimally invasive biosensing, which our current intraoperative platform may help to inform.

## 5. Conclusions

In conclusion, the device developed in this study offers a safe, practical, and objective method for characterizing the mechanical properties of the eye. This system has the potential to improve diagnostic accuracy and support more personalized, data-guided treatment for patients with ocular motility disorders.

## Data Availability

The datasets used and/or analyzed during the current study are available from the corresponding author upon reasonable request.

## Abbreviations

The following abbreviations are used in this manuscript:

FDT	Forced Duction Test
PCB	Printed Circuit Board
IR	Infra-Red
